# Improved Long-Term Imaging of Embryos with Genetically Encoded α-Bungarotoxin

**DOI:** 10.1371/journal.pone.0134005

**Published:** 2015-08-05

**Authors:** Ian A. Swinburne, Kishore R. Mosaliganti, Amelia A. Green, Sean G. Megason

**Affiliations:** Department of Systems Biology, Harvard Medical School, Boston, Massachusetts, United States of America; Peking University, CHINA

## Abstract

Rapid advances in microscopy and genetic labeling strategies have created new opportunities for time-lapse imaging of embryonic development. However, methods for immobilizing embryos for long periods while maintaining normal development have changed little. In zebrafish, current immobilization techniques rely on the anesthetic tricaine. Unfortunately, prolonged tricaine treatment at concentrations high enough to immobilize the embryo produces undesirable side effects on development. We evaluate three alternative immobilization strategies: combinatorial soaking in tricaine and isoeugenol, injection of α-bungarotoxin protein, and injection of α-bungarotoxin mRNA. We find evidence for co-operation between tricaine and isoeugenol to give immobility with improved health. However, even in combination these anesthetics negatively affect long-term development. α-bungarotoxin is a small protein from snake venom that irreversibly binds and inactivates acetylcholine receptors. We find that α-bungarotoxin either as purified protein from snakes or endogenously expressed in zebrafish from a codon-optimized synthetic gene can immobilize embryos for extended periods of time with few health effects or developmental delays. Using α-bungarotoxin mRNA injection we obtain complete movies of zebrafish embryogenesis from the 1-cell stage to 3 days post fertilization, with normal health and no twitching. These results demonstrate that endogenously expressed α-bungarotoxin provides unprecedented immobility and health for time-lapse microscopy.

## Introduction

Recent advances in microscopy and genetic labeling techniques have made it possible to accurately track developmental events through time with microscopic resolution in live embryos [1–3]. Accurate tracking of microscopic objects (e.g. individual cells) during development enables cellular dynamics and regulatory networks to be analyzed quantitatively, illuminating previously obscured principles of development [4–9]. However, starting with the zebrafish’s first spontaneous twitches at 18 hours post fertilization (hpf), motility of the developing embryo is a challenging obstacle to long-term live imaging [10]. To achieve accurate tracking, one must be able to uniquely identify a feature of interest throughout a series of time points. When features of interest are many and crowded, tracking between time points becomes challenging to impossible if interrupted by an organism’s movement. Additionally, a feature can no longer be tracked if movement causes it to leave the field of view or become too blurred. Long-term (i.e. hours to days) live imaging of zebrafish with accurate tracking of microscopic features necessitates an effective method for long-term, healthy immobilization.

The anesthetic tricaine is the basis of current techniques for immobilizing zebrafish during long-term imaging [11]. Preferential use of tricaine for zebrafish research is likely a holdover from it being the only FDA approved anesthetic for aquaculture. Tricaine immobilizes zebrafish by blocking sodium action potentials, thus preventing muscle contractions [12, 13]. Tricaine, previously used for short-term immobilization of adults, has been co-opted for long-term immobilization of embryos.

Embryos intermittently anesthetized with 30 μg/ml tricaine between 20 and 48 hpf appear 2–6 hours younger than control embryos [10]. Tricaine concentrations as low as 100 μg/ml can cause pericardial edema even without complete immobilization [11]. To what extent this edema is an indicator of more general toxicity or developmental defects is unclear. Tricaine also slows the heart rate and alters developmental mechanisms dependent on hemodynamics such as heart and vascular development [14]. Herein we present an additional toxicity to the development of the embryo: prolonged exposure to tricaine causes reduced growth of the otic vesicle and impairment of semicircular canal development.

Modern clinical anesthesia combines analgesics and anesthetics to achieve the desired anesthetic end point [15]. Combinatorial anesthetics allow greater control of: target state duration, speed to target state, severity of side effects, and comfort of recovery. This strategy takes advantage of the fact that many anesthetics have mechanisms that are different but co-operate towards a desired target state whereas the side effect mechanisms do not co-operate. For example, combined usage of tricaine and isoflurane on zebrafish can minimize short-term side effects on cardiac rhythm [16]. We chose to test whether tricaine co-operates with a second anesthetic, isoeugenol, which has previously shown utility in zebrafish [17, 18] and appears to act through a different mechanism to tricaine, namely inhibition of voltage-gated calcium channels, voltage-gated sodium channels, and nicotinic receptors in muscle [19–22]. Here we report that combining tricaine and isoeugenol can result in better long-term imaging than monotherapy, although health is still an issue.

Due to the range of side effects often seen with anesthetics, we explored other methods for immobilization. α-bungarotoxin is a neurotoxic protein isolated from a venomous snake, the Taiwan banded krait [23]. α-bungarotoxin causes paralysis through irreversible binding of nicotinic acetylcholine receptors (AChR alpha 1 and 7 subunits). It is widely used for short-term neurophysiological recording since anesthetics can alter neural activity [24–26]. α-bungarotoxin has not previously been explored in the context of long-term immobilization nor has it been successfully expressed within a heterologous system with the goal of having an organism paralyze itself. Commercially available α-bungarotoxin is currently harvested from the venom of poisonous snakes rather than made recombinantly raising the possibility that factors such as poor translation, lack of cofactors, improper folding, improper glycosylation, or inefficient secretion could abolish its activity when made heterologously. Despite the concerns, we find that α-bungarotoxin, via protein injection or more conveniently and flexibly via endogenous expression from a synthetic codon-optimized gene, results in unprecedented level of immobility for long-term imaging while preserving embryonic health.

## Materials and Methods

### Ethics statement

Zebrafish work was approved by the Harvard Medical Area Standing Committee on Animals under protocol number 04487.

### Zebrafish mobility

Embryos were poked repeatedly with a hair loop while monitoring tail movement under a dissecting microscope (adapted from [27]). Individual embryos were scored as immobile only if they failed to twitch in response to ~5 pokes. 10–30 embryos were tested for each condition and time point.

### Health measurements

**Fig 1:**
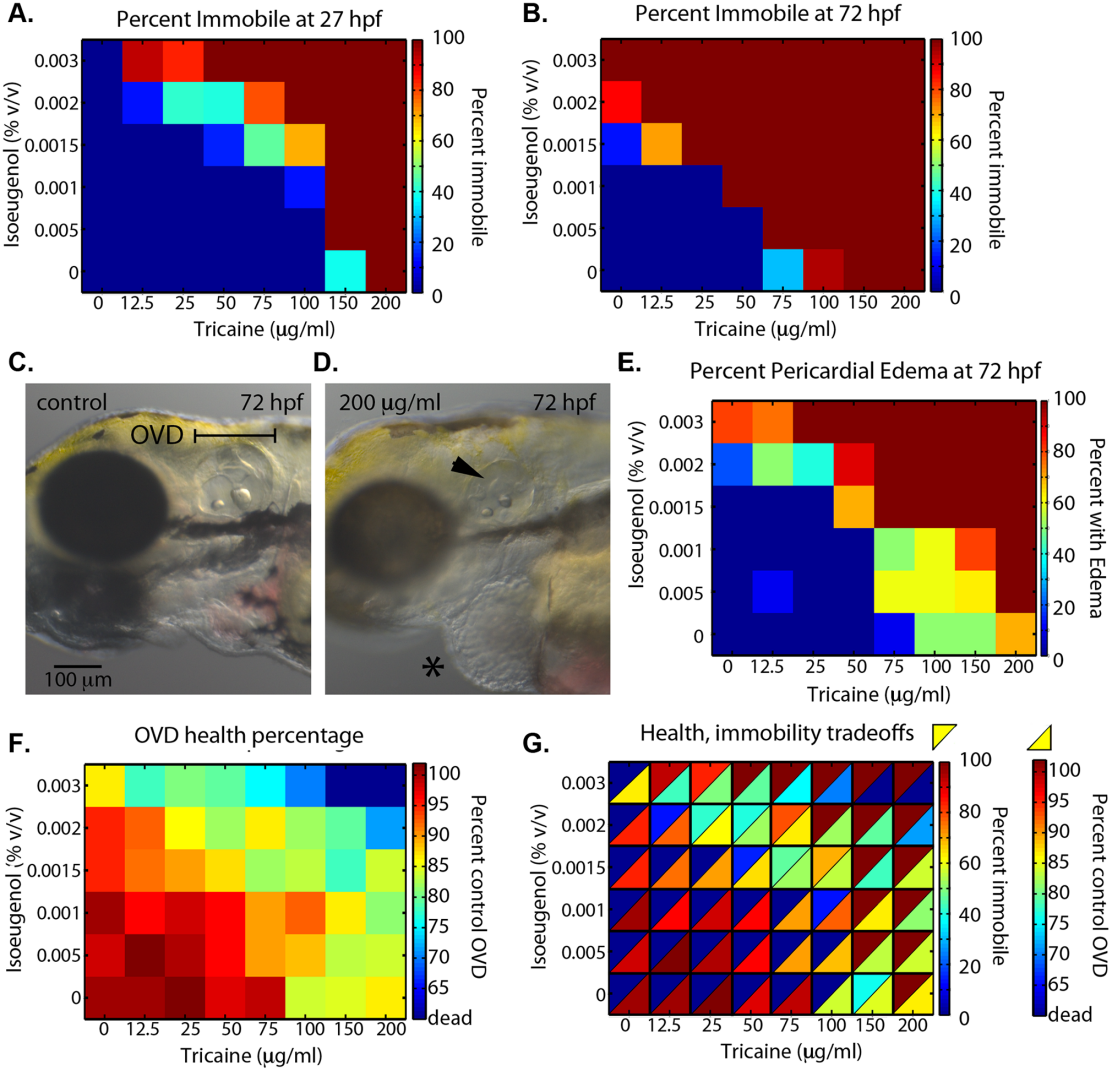
Tricaine and isoeugenol co-operate towards healthier immobilization. **(A)** Heat map of percent immobile for 48 combinations of tricaine (0–200 μg/ml) and isoeugenol (0–0.003% v/v). Embryos were dechorionated and soaked from 24–27 hpf when they were assayed for immobility. **(B)** Continuation of treatments from (A), embryos were assayed for immobility at 72 hpf. **(C,D)** Representative micrographs of control (C) and 200 μg/ml tricaine treated (D) embryos. Arrow in (D) shows failure of semicircular canal projection fusion. Asterisk in (D) shows pericardial edema. **(E)** Heat map of percent of embryos with pericardial edema at 72 hpf. **(F)** Percent control otic vesicle diameter (OVD) was calculated by dividing the average of 10–30 experimental embryos by the average of 10–30 control embryos. Heat map of percent control OVD for the combinatorial treatments. OVD was measured at 72 hpf using micrographs like those in (C, D). Percentage is based on normalization to untreated control. **(G)** Merge of heatmaps from (A, 27 hpf) and (F) that highlights the tradeoffs between embryo immobility and healthy development.

Embryo length, pericardial edema, and otic vesicle diameter (OVD) were determined at 72 hpf. Images of embryos and a stage micrometer (Ward’s) were captured with an Olympus MVX10 Macroview (Olympus) using either a 1x MVX plan apochromat, 0.25NA objective for 25x magnification or a 2x MVX plan apochromat, 0.5NA for 126x magnification. Embryo features were measured using ImageJ. OVD was measured at 72 hpf using micrographs like those in (Fig 1C and 1D). Percentage is based on normalization to controls.

### Embryo treatments

Starting at 24 hpf, embryos were soaked in Danieau buffer containing 48 combinations of 0–200 μg/ml tricaine (Sigma Aldrich) and 0–0.003% v/v isoeugenol (Sigma Aldrich). A zebrafish codon optimized α-bungarotoxin ORF was synthesized (GeneArt/ Life Technologies) and sub-cloned into a construct with a T7 promoter at the 5’ end (pMTB-T7- α-bungarotoxin). The sequence is presented in the S1 Text and is available in GenBank (accession number KT279887). α-bungarotoxin mRNA was synthesized from a linearized plasmid using the mMessage mMachine T7 ULTRA kit (Invitrogen). Subsequently, mRNA was purified using RNAeasy Mini Kit (Qiagen). α-bungarotoxin protein was obtained from Tocris. 3 kDa dextran-Texas red and Alexa-Fluor 594 conjugated α-bungarotoxin were obtained from Invitrogen. 2.3 nl injections were performed using Nanoject II (Drummond).

### Blood flow analysis

Blood flow analysis by laser-scanning velocimetry was adapted from previously described methods[28]. 72 hpf Embryos were placed in a 35 mm CELLview dish with a number 1.5 glass bottom for microscopy (Greiner Bio-One). Embryos immobilized with α-bungarotoxin mRNA were placed in Danieau buffer at 20°C. Control and tricaine treated embryos were transiently immobilized in 100 μg/ml tricaine in Danieau buffer at 20°C for 3–10 minutes. Line scanning was performed using the confocal DIC mode on an inverted Nikon A1R point scanning confocal using a 60x Plan Apo 1.40 NA oil immersion lens and a 488 nm argon-krypton laser. Lines acquired parallel with the dorsal aorta, above the yolk, were 107.52 μm long (512 pixels at 0.21 μm per pixel) and were acquired continuously with a resonant scanner so that the time step was 0.126 ms per line.

### Automated quantification of blood velocities

To reconstruct instantaneous velocities of cells suspended in moving plasma, we developed a fully-automated, open-source, image-analysis pipeline in C++ using the Insight Toolkit (ITK) libraries (www.itk.org). While similar to previous strategies, our scripts fully-automate image analysis and incorporate information from the entire image[28]. The analysis software is available upon request and will be described in detail elsewhere.

### Long-term imaging

The 85 hour α-bungarotoxin movie was acquired on an Olympus MVX10 (Olympus) within a home-made foam core chamber heated to 28.5°C and humidified with a large beaker of standing water. See S1 and S2 Figs, S1 Movie, and S1 Text for details. The movie is also available at http://www.youtube.com/watch?v=4c-Kw4timVA&feature=youtu.be. Additional movie is available at https://www.youtube.com/watch?v=A1vun3ETAkE.

## Results and Discussion

### Combinatorial anesthesia

Our first approach to long-term immobilization combined tricaine and isoeugenol. Like clinicians, we reasoned that the two anesthetics have different targets and different side effects; therefore, they may co-operate toward the desired immobilization while minimizing toxicity. This approach was attractive because both anesthetics are inexpensive, with histories of efficacy and low toxicity in fish. Additionally, this approach would be non-invasive and allow the pre-screening of embryos for desired phenotypes.

We first collected embryos from crosses of the wild-type line AB. At 24 hpf we immersed dechorionated embryos (~10–20 per condition) in one of 48 different combinations of tricaine (0 to 200 μg/ml) and isoeugenol (0 to 0.003% v/v) prepared in Danieau buffer (Fig 1A). The zebrafish touch response first develops between 24 and 27 hpf [10]. At 27, 30, 48, 54, and 72 hpf we scored embryos for mobility (27 and 72 hpf, Fig 1A and 1B, S3 Fig). We scored embryos as immobile when they did not move in response to several firm pokes. In our experience of long-term imaging after 27 hpf, non-responsiveness of the embryo to firm poking correlates well with immobility throughout time courses lasting several days.

Tricaine and isoeugenol co-operate towards the desired anesthetic endpoint of embryo immobility (Fig 1A). For all embryos to be completely immobile at 27 hpf requires 200 μg/ml of tricaine on its own. The highest concentration of isoeugenol we tested, 0.003%, was unable to immobilize the embryos until 30 hpf. However, 20 combinations of lower concentrations of tricaine and isoeugenol were able to immobilize all or at least some fraction of the total count of embryos (Fig 1A). Together these results indicate that tricaine and isoeugenol can work together to immobilize embryos.

Embryo sensitivity for reaching the immobilization endpoint with these anesthetics increased during the second and third days of development. We continued to track mobility of embryos in the 48 conditions over the second, third, and fourth days of development. We found more embryos immobilized at each time point (Fig 1A and 1B, S3A–S3E Fig). For instance, the combination of 50 μg/ml tricaine and 0.001% isoeugenol did not immobilize any embryos until 54 hpf (Fig 1A and 1B and S3A–S3E Fig). We confirmed that this trend of increasing sensitivity does not require the prior day of soaking as this combination of anesthetics induces immobilization in 12/19 embryos when soaking began at 54 hpf. We conclude that dosage should be optimized to the period of development when using anesthetics to immobilize embryos, and embryos are most difficult to immobilize around 24 hpf.

**Fig 2:**
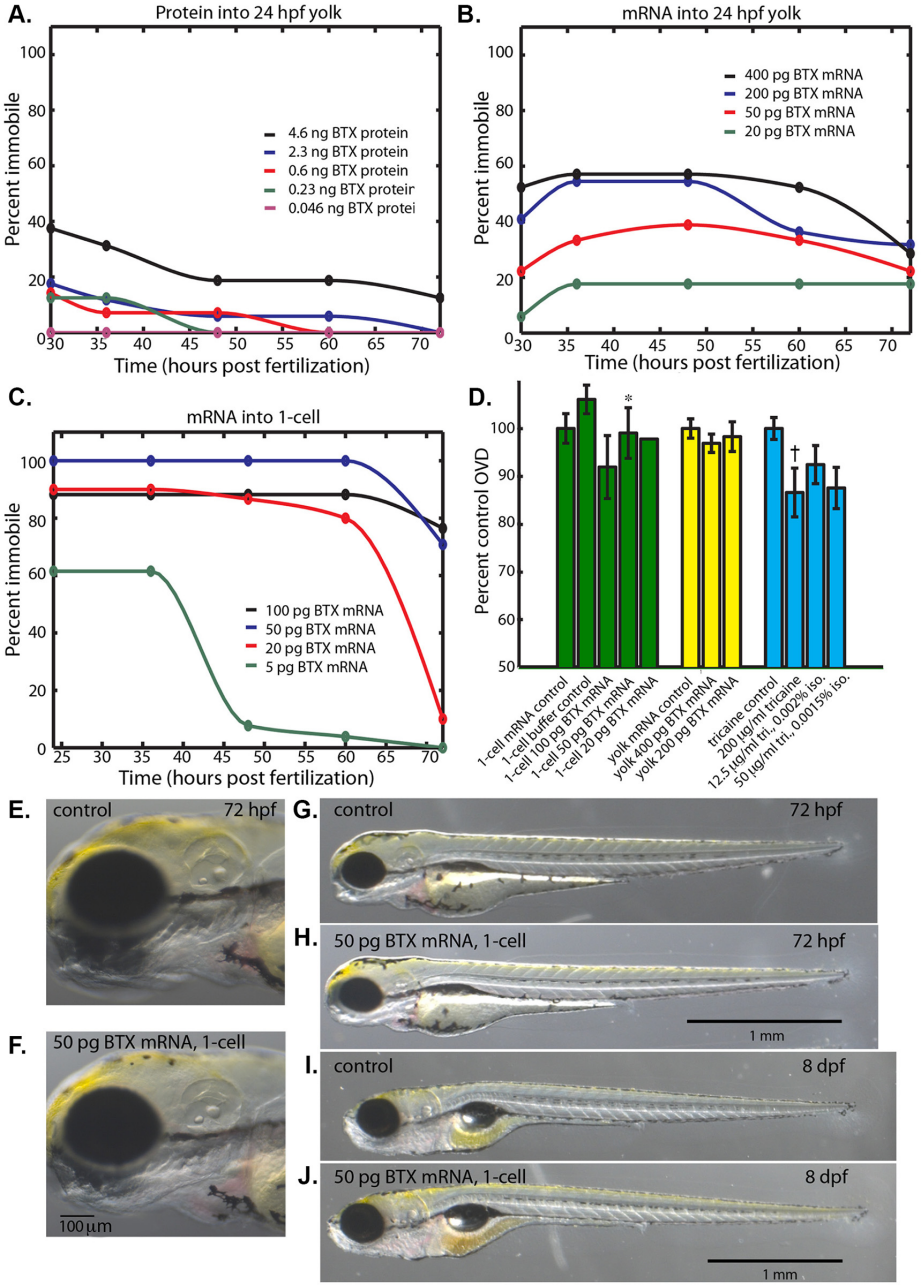
α-bungarotoxin immobilizes embryos while permitting normal development. **(A)** Percent of embryos immobile after injection of α-bungarotoxin protein (0.046–4.6ng) into the yolk at 24 hpf. **(B)** Percent of embryos immobile after injection of α-bungarotoxin mRNA (20–400 pg) into the yolk at 24 hpf. **(C)** Percent of embryos immobile after injection of of α-bungarotoxin mRNA (5–100 pg) into the 1-cell zygote. **(D)** Percent control OVD at 72 hpf for injection of α-bungarotoxin mRNA into the 1-cell zygote (green), into the yolk (yellow), and reference anesthetic treatments that permitted long-term immobilization (blue). (*) Not significantly different from control, Mann-Whitney-Wilcoxon two tailed P-value 0.87. (†) Significantly different from control, Mann-Whitney-Wilcoxon two tailed P-value 0.0011. **(E, G)** Control embryo at 72 hpf that was injected with 50 pg of membrane-citrine mRNA into the 1-cell zygote. **(F, H)** 72 hpf embryo that was injected with 50 pg of α-bungarotoxin mRNA into the 1-cell zygote. **(I)** Control larva at 8 days post fertilization (dpf) injected with 50 pg of membrane-citrine mRNA into the 1-cell zygote. **(J)** 8 dpf larva that was injected with 50 pg of α-bungarotoxin mRNA into the 1-cell zygote.

Combinations of tricaine and isoeugenol can minimize toxicity while still achieving immobility. We first examined heart edema as a read-out of embryo health and found it to be sensitive to both tricaine and isoeugenol (Fig 1E). We then focused on the size of an embryo’s otic vesicle as an indicator of developmental progress and health, because its morphogenesis begins early and is easily measured. Also, as illustrated here, reduced otic vesicle size correlates well with other health indicators such as pericardial edema while being a more sensitive and continuous readout of health (Fig 1E and 1F). Reduced otic vesicle size could be because of reduced lumen expansion or reduced proliferation. Tricaine and isoeugenol both act through ion channels in ways that could directly reduce lumen expansion. While the specific target of tricaine is not known, the *scna* gene family is believed to mediate a large portion of sodium conductance in zebrafish and some may be expressed in the developing otic vesicle [29, 30]. However, it is unclear if any are expressed in the developing heart so there are likely to be other targets that cause tricaine’s side effects. Additionally, off-target effects or overall poor health could reduce proliferation or indirectly influence complex morphogenetic processes. At 72 hpf we measured the diameter of the left otic vesicle along the anterior-posterior axis (Fig 1C, Otic Vesicle Diameter, OVD). Among 10 control embryos the OVD is 164 μm ± 4.0 μm (mean ± standard deviation; representative control embryo, Fig 1C). When soaked in 200 μg/ml of tricaine from 24 to 72 hpf the average OVD was significantly shorter at 148 ± 8.7 μm (representative embryo treated with 200 μg/ml tricaine, Mann-Whitney-Wilcoxon two tailed P-value < 0.0011, Figs 1D and 2D). We also observed an increased incidence of pericardial edema (asterisk, Fig 1D) and impaired tissue fusion during semicircular canal development (arrowhead, Fig 1D).

We set out to determine whether a combination of tricaine and isoeugenol could reduce toxicity while still achieving immobility by measuring OVD at 72 hpf for all 48 conditions (Fig 1F). We found some additive toxicity supported by a gradual decrease in OVD as the combination of tricaine and isoeugenol increased. For example, when treated with 150 μg/ml of tricaine on its own the average OVD was 85% of the control. However, the average OVD decreased to 80% when 150 μg/ml of tricaine was combined with 0.002% isoeugenol. We observed a similar trend when scoring embryos for pericardial edema at 72 hpf (Fig 1E). All embryos died when treated with 150 μg/ml tricaine plus 0.003% isoeugenol. Our idealized criteria for acceptable immobilization would be long-term immobility without significant reduction in OVD length. By integrating immobilization information with OVD length, we found two optimal combinations for long-term imaging beginning at 24 hpf: 12.5 μg/ml tricaine and 0.002% isoeugenol or 100 μg/ml tricaine and 0.001% isoeugenol (Fig 1G). With these combinations, one can study those few embryos that are immobilized on the first day (Fig 1A, 12–13% of embryos immobile) while maintaining an endpoint OVD that is ~92% that of a healthy embryo. However, for 12.5 μg/ml tricaine and 0.002% isoeugenol or 100 μg/ml tricaine and 0.001% isoeugenol this reduction in ear size was still significantly different with respect to controls (the differences are statistically significant with respective Mann-Whitney-Wilcoxon two tailed P-values of 0.007 and < 3.2e-4). In summary, none of the combinations achieved complete immobilization with statistically significant, healthy development (note the absence of immobility with healthy OVD in Fig 1G).

### α-bungarotoxin for healthy, long-term immobilization

Dissatisfied that even a minimal combination of tricaine and isoeugenol affects the health of the developing embryo, we tried a second set of long-term immobilization strategies using α-bungarotoxin. We compared three ways of delivering α-bungarotoxin: injection of protein into the yolk, such that it enters the blood stream, at 24 hpf; injection of mRNA into the yolk at 24 hpf; and injection of mRNA into the 1 cell embryo. The rationale for injecting mRNA into the yolk was that the yolk syncytial layer (YSL) remains contiguous with the yolk and would translate the mRNA. We expected that these cells would produce and secrete α-bungarotoxin into the yolk, from which it would then travel to the rest of the embryo to induce paralysis. Similar YSL targeting strategies have been used for locally restricting knock down of gene function [31].

Injecting α-bungarotoxin protein into the yolk at 24 hpf was not very efficient at inducing paralysis. It was unclear how to deliver the protein so we asked whether material injected into the yolk would be distributed throughout the embryo. We compared injections into the dorsal and ventral sides of the yolk at 24 hpf for 2.3 ng of Alexa-Fluor 594 conjugated α-bungarotoxin or 2.3 ng of 3 kDa dextran-Texas red (S4A Fig). When targeting the ventral yolk, 10 out of 10 embryos injected with either 3 kDa dextran-Texas red or Alexa-Fluor 594 conjugated α-bungarotoxin resulted in distribution of the fluorescent material throughout the embryo in a manner that suggests entry into the circulatory system (S4C–S4F Fig). When targeting the dorsal yolk, 9 out of 9 dextran-Texas red and 8 out of 8 Alexa-Fluor 594 conjugated α-bungarotoxin injections resulted in distribution of the fluorescent material throughout the embryo in a manner that suggests entry into the circulatory system (S4G–S4J Fig). Confocal imaging of an embryo’s yolk after injection with 2.3 ng of Alexa-Fluor 594 cojugated α-bungarotoxin showed fluorescent signal surrounding the yolk that was continuous with the fluid entering the heart (S4B Fig). This suggests that material injected into the yolk enters the blood stream via this peripheral yolk space. For long-term analysis of protein injections, we explored a range between 0.046 ng and 4.6 ng of unlabeled α-bungarotoxin protein. Similar amounts were previously used in α-bungarotoxin protein injection strategies where a few nanoliters of 125 μM α-bungarotoxin were injected into the embryonic heart (125 μM is ~1ng/nl of α-bungarotoxin) [26]. When we injected 4.6 ng of protein into the ventral yolk of 16 embryos at 24 hpf, only 6 were immobilized at 30 hpf (Fig 2A). Of these 6 paralyzed embryos, only 2 remained paralyzed 48 hours later. Protein injection into the yolk may work inconsistently because of variable pharmokinetics from being trapped, aggregated, denatured, or degraded in the yolk.

Injecting mRNA into the 24 hpf yolk was slightly more efficient and persistent than protein injection. To produce α-bungarotoxin mRNA we had the gene synthetized with codons optimized for zebrafish. Using in vitro transcribed mRNA produced by standard molecular biology techniques, we injected various amounts of mRNA into the yolk. We presumed that accessible YSL cells would take up mRNA, express protein, and secrete the toxin to the rest of the embryo. This strategy worked better than injection of protein into the yolk (Fig 2B). For instance, injection of 400 pg of mRNA into 22 embryos at 24 hpf caused 12 to be paralyzed at 36 hpf. Of these 12, only 7 remained paralyzed 48 hours later. This was an indication that the paralysis induced by injection of α-bungarotoxin mRNA into yolk is slightly more efficient and persistent than the protein injections.

Injecting mRNA into the 1-cell embryo works very well for efficient paralysis induction and persistence of paralysis. Specifically, injection of 50 pg of α-bungarotoxin mRNA into 24 embryos at the 1-cell stage caused all 24 to be paralyzed at 24 hpf (Fig 2C). 24 out of 24 remained paralyzed at 60 hpf, while only 7 of the 24 began to respond to poking at 72 hpf. Persistence of immobility to 72 hpf decreased with concentration of mRNA (persistence fractions of 0.86, 0.70, 0.11, and 0 for 100, 50, 20, and 5 pg of mRNA). This relationship suggests that the limit on the persistence of paralysis is the degradation of the α-bungarotoxin mRNA and protein.

Injection of α-bungarotoxin mRNA is less detrimental to development than soaking embryos in anesthetic. In contrast to embryos immobilized with anesthetics we never observed pericardial edema in embryos immobilized with α-bungarotoxin. We measured OVD at 72 hpf of the embryos injected with α-bungarotoxin mRNA into the 1-cell zygote and in the yolk at 24 hpf (Fig 2D). Except for the highest dose of mRNA injected into the 1-cell stage (100 pg), the various mRNA injections caused no significant reduction in OVD when compared to controls (injection of an mRNA encoding a membrane targeted citrine fluorescent protein). Indeed, embryos paralyzed with 50 pg of mRNA injected into the 1-cell morphologically resemble control, uninjected embryos (Fig 2E–2H, OVD not significantly different from control embryos, Mann-Whitney-Wilcoxon two tailed P-value 0.87). Beginning on the third day of development and onward, paralyzed fish begin recovering their mobility. To further explore the health of embryos injected with α-bungarotoxin mRNA, we examined larvae at 8 days post-fertilization and observed no gross defects when compared to both control and unperturbed wild type larvae (Fig 2I and 2J).

**Fig 3:**
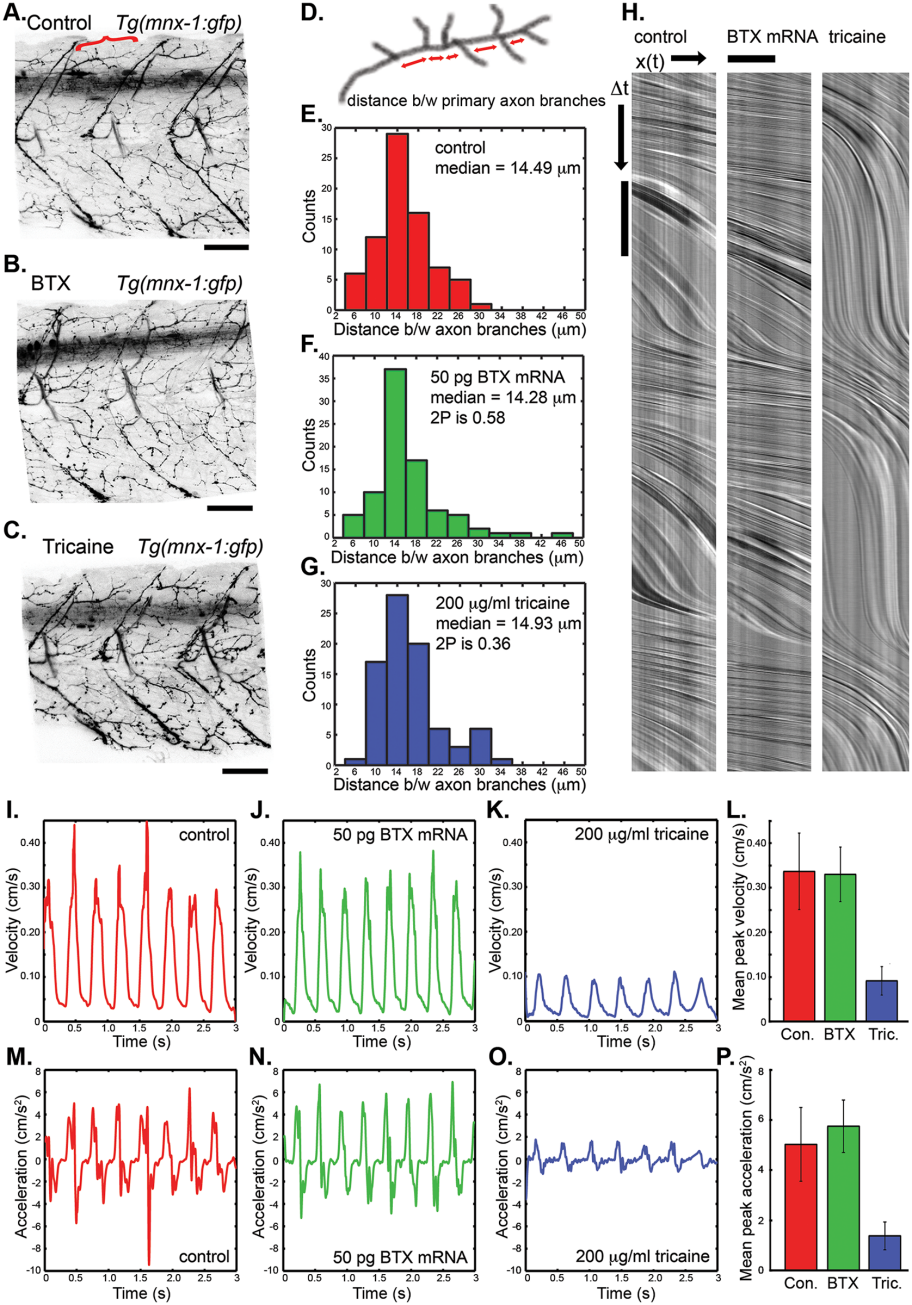
Prolonged immobilization with α-bungarotoxin mRNA does not grossly alter neural or cardiovascular development. **(A-C)** 3D reconstructions of confocal images of 72 hpf *Tg(mnx1*:*gfp)* centered at the sixth somite reveal no gross abnormalities in motor neuron patterning or development when embryos are immobilized with 50 pg of α-bungarotoxin mRNA injected at the 1-cell stage **(B)** or 200 μg/ml of tricaine from 24 to 72 hpf **(C)** (scale bar 50 μm). A stereotyped axon (red bracket, **A**, schematic, **D**) was used to quantify axon branching. **(E-G)** The distributions of distances between axon branches were not significantly altered in embryos immobilized with 50 pg of α-bungarotoxin mRNA injected at the 1-cell stage **(F,** Mann-Whitney-Wilcoxon two tailed P-value 0.58**)** or 200 μg/ml of tricaine from 24 to 72 hpf **(G,** Mann-Whitney-Wilcoxon two tailed P-value 0.36**)**. **(H)** Representative laser-scanning velocimetry results reveal no gross difference in cardiovascular performance between control embryos and embryos immobilized with 50 pg of α-bungarotoxin mRNA. 200 μg/ml of tricaine from 24 to 72 hpf does grossly alter blood flow. Vertical scale bar 100 ms, horizontal scale bar 20 μm. **(I-P)** Quantitative image analysis of laser-scanning velocimetry reveals no significant difference in cardiovascular function between control and α-bungarotoxin mRNA injected embryos while prolonged tricaine treatment significantly reduces peak blood velocity and peak blood acceleration (Mann-Whitney-Wilcoxon two tailed P-values < 1e-12, tricaine relative to control peak velocities, and <1.4e-11, tricaine relative to control peak accelerations).

α-bungarotoxin inhibits postsynaptic activation of AChR’s by binding either the alpha1 or alpha7 subunits and could potentially interfere with developmental mechanisms that may require this activation. To help determine whether prolonged expression of α-bungarotoxin protein interferes with the path finding of motor neurons we imaged *Tg(mnx1*:*gfp)* embryos at 72 hpf that had been injected with a control or α-bungarotoxin mRNA at the 1-cell stage (Fig 3A–3C) and observed no obvious differences in the motor neuron patterning or path finding. We quantified distances between branches of a stereotyped dorsal axon (red bracket in Fig 3A, schematic in Fig 3D) and found no statistical difference between the distribution of axon branches between controls, embryos injected with 50 pg of α-bungarotoxin mRNA at the 1-cell stage, and embryos treated with 200 μg/ml Tricaine from 24 to 72 hpf (Mann-Whitney-Wilcoxon test, respective two tailed P-values of 0.58 and 0.36 indicate no significant differences between axon branch distributions relative to the control). This is consistent with the ability of paralyzed embryos to rapidly recover normal motility and the lack of muscle defects. This is also consistent with the *nic1* mutant (AChR alpha 1 subunit) that lacks detectible motor neuron innervation defects or neuromuscular phenotypes [32]. However, there remains the possibility that these analyses miss more subtle differences arising from reduced feedback that can modulate synapse remodeling [33, 34]. Additionally, the subset of CNS neurons that use the AChR alpha 7 subunit could also be affected. Because α-bungarotoxin may have unidentified side effects, care should be taken when using α-bungarotoxin to study processes related to plasticity, wiring, and dynamics of neural networks.

To determine whether α-bungarotoxin alters development or physiology of the cardiovascular system we used laser-scanning velocimetry to measure blood flow velocities and accelerations at 72 hpf [28]. Representative line scans in the direction of blood flow through the dorsal aorta from control embryos transiently immobilized with 100 μg/ml tricaine and embryos injected with 50 pg of α-bungarotoxin mRNA in the 1 cell stage reveal no obvious difference between controls and embryos immobilized with α-bungarotoxin (Fig 3H). In contrast, embryos treated with 200 μg/ml of tricaine between 24 and 72 hpf exhibited severely reduced blood velocity and heart rate (Fig 3H, ~30% of embryos presented with measurable blood flow, Fig 1E). We improved the quantification of this technique by automating image analysis. Quantification reveals that heart rate, peak blood flow velocities and accelerations at 72 hpf were similar in controls and embryos injected with 50 pg of α-bungarotoxin mRNA (respective mean heart rates of 121 and 135 bpm, respective mean peak velocities of 0.34 and 0.33 cm/s, respective mean peak accelerations 5.0 and 5.7 cm/s^2^, Fig 3I, 3J, 3L, 3M, 3N and 3P). In contrast, prolonged 200 μg/ml tricaine treatment caused significantly reduced heart rate, blood velocity and blood acceleration (mean heart rate 110 bpm, Mann-Whitney-Wilcoxon two tailed P-Value < 0.0013, mean velocity 0.09 cm/s, Mann-Whitney-Wilcoxon two tailed P-value < 1e-12, mean acceleration 1.38 cm/s^2^, Mann-Whitney-Wilcoxon two tailed P-value <1.4e-11). In summary, prolonged immobilization with 50 pg of α-bungarotoxin mRNA injected at the 1 cell stage does not appear to alter the cardiovascular development or physiology as opposed to tricaine that significantly alters blood flow dynamics.

### α-bungarotoxin mRNA into the 1-cell embryo blocks first twitches

**Fig 4:**
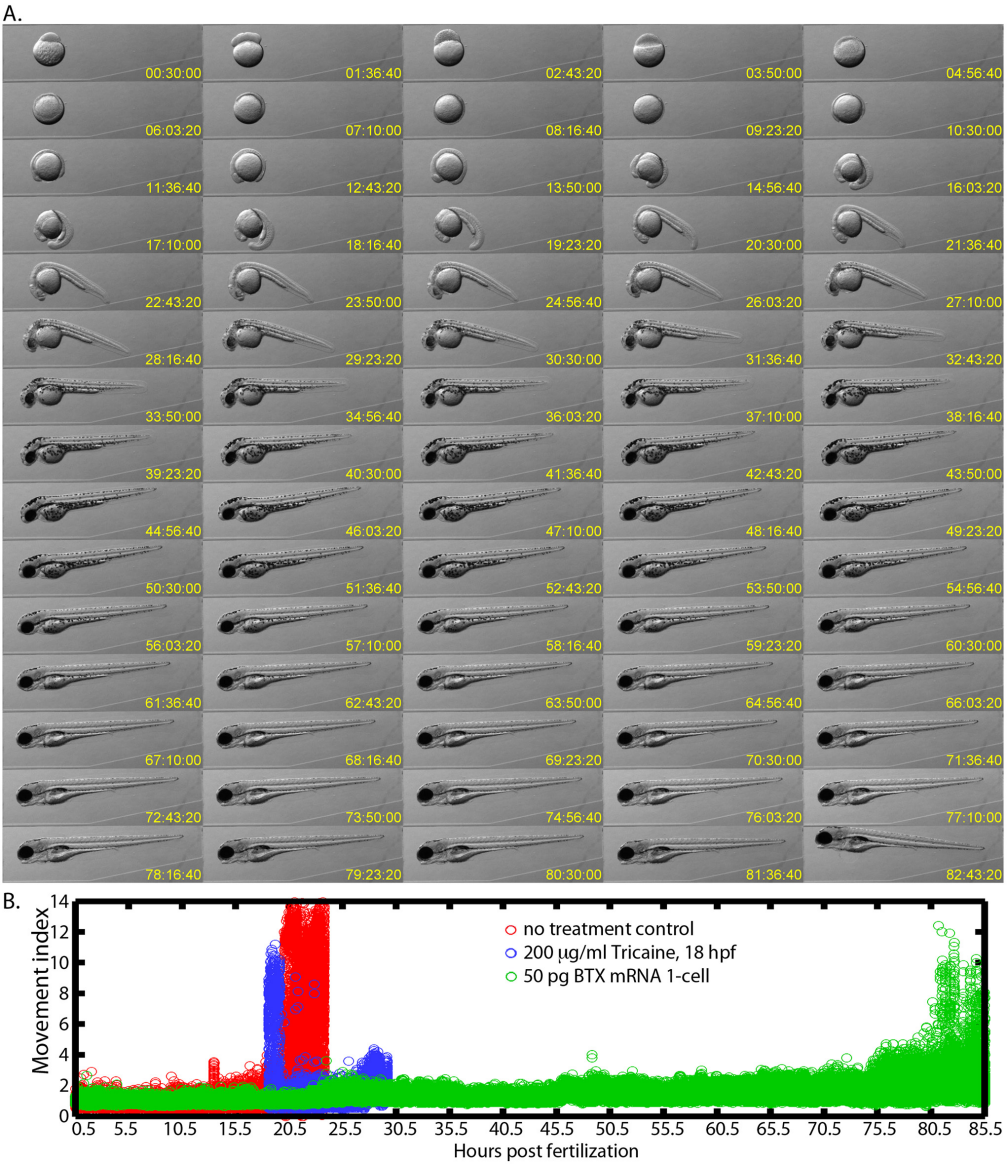
Long-term imaging of embryos immobilized with α-bungarotoxin mRNA. **(A)** Montage of an immobilized embryo’s development from the 1-cell stage to 85 hpf after it had been injected with 50 pg of α-bungarotoxin mRNA into the 1-cell. Images are shown from every hour of development. **(B)** Quantification of the full time-course that included 153,452 images that were acquired every 2 seconds. The movement index was calculated as the maximum difference between each image and its subsequent image in the time-series. The index was normalized to the average maximum difference in the first 2,000 time points. Control embryos (red) and embryos in 200 μg/ml tricaine (blue) begin twitching at around 18 hpf and then may swim out of the field while α-bungarotoxin injected embryos (green) showed very little movement until 80 hpf.

Before the touch response develops, spontaneous twitching initiates around 18 hpf. As a proof-of-principle of the 1-cell stage α-bungarotoxin mRNA injection immobilization strategy, we imaged embryonic development from the 1-cell stage to 85 hpf, for injection controls and for 50 pg of α-bungarotoxin mRNA into the 1-cell (Fig 4A). Images were acquired every 2 seconds but only presented for every hour in Fig 4A, or every 40–160 seconds in S1 Movie. We quantified movement by calculating the maximum pixel intensity difference between successive time-points within the full data-set (2 second time resolution, Fig 4B). Notably, α-bungarotoxin mRNA injected embryos do not perform the first twitches at 18 hpf or anytime thereafter, allowing us to capture an unprecedentedly complete movie (Fig 4B, S1 Movie). In contrast, control embryos and embryos treated with 200 μg/ml tricaine still began to move at 18 hpf (Fig 4B). What movement can be observed in the α-bungarotoxin time-course and its quantification is minor and includes: embryo rolling during cleavage and epiboly (0.5–5.5 hpf), embryo slippage within the agarose mount during tail extension (~24 hpf and ~47 hpf), jaw movement (starting ~ 75 hpf), and recovery from paralysis (starting ~80 hpf). A movie is here as S1 Movie and is available at http://www.youtube.com/watch?v=4c-Kw4timVA&feature=youtu.be. An additional movie is available at https://www.youtube.com/watch?v=A1vun3ETAkE. We have imaged 9 embryos injected with 50 pg of BTX mRNA and all have lacked movement until >72 hpf. Thus, injection of α-bungarotoxin mRNA at the 1-cell stage allows the tracking of normally developing embryos through long periods of time including the period of first twitches, which were typically difficult to stop with previous immobilization approaches.

The results presented here demonstrate versatile strategies for immobilizing the zebrafish embryo for extended periods of time. Combinations of tricaine and isoeugenol can reduce toxicity while still achieving an immobile anesthetic endpoint. Injection of α-bungarotoxin mRNA causes paralysis of the embryo that persists for days while healthy development continues. These novel strategies for embryo immobilization will enable accurate, long-term studies of healthy fish that were not previously possible using tricaine alone. Analogous to GFP, the ability to use genetically encoded α-bungarotoxin rather than just exogenous protein may permit much more versatile, precise, and flexible approaches. Similar approaches may also find utility for long-term time-lapse imaging in other model organisms.

## Supporting Information

S1 Fig Blueprints for foam core incubator. Panels were assembled by gluing one side of each hinge to a panel (locations depicted by dash rectangles containing ‘h’) and secured to the corresponding neighbor panel with nuts and screws inserted into holes drilled in foam core panels and hinges (depicted as black dots).
(TIF)

S2 Fig Schematic of Lucite mold for generating lateral mounts in agarose used for long-term imaging and image of skylight petri lid. **(A)** Mold design customized so that embryo’s yolk sits in dimpled depression and tail can extend along a graded ramp of agarose. The graded ramp maintains more of the fish in the same focal plane. **(B)** Image of “skylight” petri dish used for long-term imaging.
(TIF)

S3 Fig Tricaine and isoeugenol co-operate towards healthier immobilization. **(A-E)** Heat maps of percent immobile for 48 combinations of tricaine (0–200 μg/ml) and isoeugenol (0–0.003% v/v). Embryos were dechorionated and soaked starting at 24 hpf. Embryos were assayed for immobility at 27, 30, 48, 54, and 72 hpf.
(TIF)

S4 Fig α-bungarotoxin protein injected into the yolk is distributed throughout the embryo. **(A)** Two injection strategies explored for α-bungarotoxin protein injection: either into the ventral side of the yolk or the dorsal side of the yolk of zebrafish embryos at 24 hpf. **(B)** Fluorescence from 2.3 ng Alexa-Fluor 594 conjugated α-bungarotoxin injected into the ventral yolk imaged by laser-scanning confocal microscopy. The peripheral yolk space appears continuous with the fluid entering the heart. DIC images (left) and fluorescent images (right) of representative embryos receiving ventral yolk injections of 2.3 ng 3 kDa dextran-Texas red **(C-D)**, ventral yolk injections of 2.3 ng Alexa-Fluor 594 conjugated α-bungarotoxin **(E-F)**, dorsal yolk injections of 2.3 ng 3 kDa dextran-Texas red **(G-H)**, and dorsal yolk injections of 2.3 ng Alexa-Fluor 594 conjugated α-bungarotoxin **(I-J)**. Scale bar in **(C)** applies for **(C-J)**.
(TIF)

S1 Movie Long-term imaging of embryos immobilized with α-bungarotoxin mRNA. Movie of an immobilized embryo’s development from the 1-cell stage to 85 hpf after it had been injected with 50 pg of α-bungarotoxin mRNA into the 1-cell. Frames are every 40 seconds of developmental time from 30 minutes to 12 hpf, every 80 seconds of developmental time from 12–24 hpf, and every 160 seconds of developmental time from 24 hpf to the completion of the movie. The compiled movie plays at 30 frames per second. Variable frame rates were used to better show morphogenetic movements that are relatively faster at earlier than later stages.
(MOV)

S1 Text Sequence of synthetic α-bungarotoxin gene and extended protocol for long-term imaging. Text file containing the sequence of the synthetic α-bungarotoxin gene. Extended protocol for long-term imaging.
(DOCX)

## References

[1] Megason SG. In toto imaging of embryogenesis with confocal time-lapse microscopy. Methods in molecular biology. 2009;546:317–32. 10.1007/978-1-60327-977-2_19 19378112 PMC2826616

[2] Megason SG, Fraser SE. Imaging in systems biology. Cell. 2007;130(5):784–95. 10.1016/j.cell.2007.08.031 . 17803903

[3] Huisken J, Swoger J, Del Bene F, Wittbrodt J, Stelzer EH. Optical sectioning deep inside live embryos by selective plane illumination microscopy. Science. 2004;305(5686):1007–9. 10.1126/science.1100035 . 15310904

[4] Xiong F, Tentner AR, Huang P, Gelas A, Mosaliganti KR, Souhait L, et al Specified neural progenitors sort to form sharp domains after noisy Shh signaling. Cell. 2013;153(3):550–61. 10.1016/j.cell.2013.03.023 23622240 PMC3674856

[5] Keller PJ, Schmidt AD, Wittbrodt J, Stelzer EH. Reconstruction of zebrafish early embryonic development by scanned light sheet microscopy. Science. 2008;322(5904):1065–9. 10.1126/science.1162493 . 18845710

[6] Dempsey WP, Fraser SE, Pantazis P. PhOTO zebrafish: a transgenic resource for in vivo lineage tracing during development and regeneration. PLoS One. 2012;7(3):e32888 10.1371/journal.pone.0032888 22431986 PMC3303793

[7] Olivier N, Luengo-Oroz MA, Duloquin L, Faure E, Savy T, Veilleux I, et al Cell lineage reconstruction of early zebrafish embryos using label-free nonlinear microscopy. Science. 2010;329(5994):967–71. 10.1126/science.1189428 . 20724640

[8] Venkiteswaran G, Lewellis SW, Wang J, Reynolds E, Nicholson C, Knaut H. Generation and dynamics of an endogenous, self-generated signaling gradient across a migrating tissue. Cell. 2013;155(3):674–87. 10.1016/j.cell.2013.09.046 24119842 PMC3842034

[9] McMahon A, Supatto W, Fraser SE, Stathopoulos A. Dynamic analyses of Drosophila gastrulation provide insights into collective cell migration. Science. 2008;322(5907):1546–50. 10.1126/science.1167094 19056986 PMC2801059

[10] Kimmel CB, Ballard WW, Kimmel SR, Ullmann B, Schilling TF. Stages of embryonic development of the zebrafish. Dev Dyn. 1995;203(3):253–310. Epub 1995/07/01. 10.1002/aja.1002030302 . 8589427

[11] Kaufmann A, Mickoleit M, Weber M, Huisken J. Multilayer mounting enables long-term imaging of zebrafish development in a light sheet microscope. Development. 2012;139(17):3242–7. 10.1242/dev.082586 . 22872089

[12] Frazier DT, Narahashi T. Tricaine (MS-222): effects on ionic conductances of squid axon membranes. European journal of pharmacology. 1975;33(2):313–7. . 171163 10.1016/0014-2999(75)90175-2

[13] Neumcke B, Schwarz W, Stampfli R. Block of Na channels in the membrane of myelinated nerve by benzocaine. Pflugers Archiv: European journal of physiology. 1981;390(3):230–6. . 6265861 10.1007/BF00658267

[14] Culver JC, Dickinson ME. The effects of hemodynamic force on embryonic development. Microcirculation. 2010;17(3):164–78. 10.1111/j.1549-8719.2010.00025.x 20374481 PMC2927969

[15] Amrein R, Hetzel W, Allen SR. Co-induction of anaesthesia: the rationale. European journal of anaesthesiology Supplement. 1995;12:5–11. . 8719664

[16] Huang WC, Hsieh YS, Chen IH, Wang CH, Chang HW, Yang CC, et al Combined use of MS-222 (tricaine) and isoflurane extends anesthesia time and minimizes cardiac rhythm side effects in adult zebrafish. Zebrafish. 2010;7(3):297–304. 10.1089/zeb.2010.0653 . 20807039

[17] Grush J, Noakes DL, Moccia RD. The efficacy of clove oil as an anesthetic for the zebrafish, Danio rerio (Hamilton). Zebrafish. 2004;1(1):46–53. 10.1089/154585404774101671 . 18248205

[18] Gladden JN, Brainard BM, Shelton JL, Camus AC, Divers SJ. Evaluation of isoeugenol for anesthesia in koi carp (Cyprinus carpio). American journal of veterinary research. 2010;71(8):859–66. 10.2460/ajvr.71.8.859 . 20673083

[19] Park CK, Li HY, Yeon KY, Jung SJ, Choi SY, Lee SJ, et al Eugenol inhibits sodium currents in dental afferent neurons. Journal of dental research. 2006;85(10):900–4. . 16998128 10.1177/154405910608501005

[20] Chung G, Rhee JN, Jung SJ, Kim JS, Oh SB. Modulation of CaV2.3 calcium channel currents by eugenol. Journal of dental research. 2008;87(2):137–41. . 18218839 10.1177/154405910808700201

[21] Lee MH, Yeon KY, Park CK, Li HY, Fang Z, Kim MS, et al Eugenol inhibits calcium currents in dental afferent neurons. Journal of dental research. 2005;84(9):848–51. . 16109996 10.1177/154405910508400913

[22] Ingvast-Larsson JC, Axen VC, Kiessling AK. Effects of isoeugenol on in vitro neuromuscular blockade of rat phrenic nerve-diaphragm preparations. American journal of veterinary research. 2003;64(6):690–3. . 12828253 10.2460/ajvr.2003.64.690

[23] Chang CC, Lee CY. Isolation of Neurotoxins from the Venom of Bungarus Multicinctus and Their Modes of Neuromuscular Blocking Action. Archives internationales de pharmacodynamie et de therapie. 1963;144:241–57. . 14043649

[24] Zhang LI, Tao HW, Holt CE, Harris WA, Poo M. A critical window for cooperation and competition among developing retinotectal synapses. Nature. 1998;395(6697):37–44. . 9738497 10.1038/25665

[25] Orger MB, Kampff AR, Severi KE, Bollmann JH, Engert F. Control of visually guided behavior by distinct populations of spinal projection neurons. Nature neuroscience. 2008;11(3):327–33. 10.1038/nn2048 18264094 PMC2894808

[26] Trapani JG, Nicolson T. Physiological recordings from zebrafish lateral-line hair cells and afferent neurons. Methods Cell Biol. 2010;100:219–31. 10.1016/B978-0-12-384892-5.00008-6 . 21111219

[27] Granato M, van Eeden FJ, Schach U, Trowe T, Brand M, Furutani-Seiki M, et al Genes controlling and mediating locomotion behavior of the zebrafish embryo and larva. Development. 1996;123:399–413. Epub 1996/12/01. . 9007258 10.1242/dev.123.1.399

[28] Malone MH, Sciaky N, Stalheim L, Hahn KM, Linney E, Johnson GL. Laser-scanning velocimetry: a confocal microscopy method for quantitative measurement of cardiovascular performance in zebrafish embryos and larvae. BMC biotechnology. 2007;7:40 10.1186/1472-6750-7-40 17623073 PMC1955438

[29] Wu SH, Chen YH, Huang FL, Chang CH, Chang YF, Tsay HJ. Multiple regulatory elements mediating neuronal-specific expression of zebrafish sodium channel gene, scn8aa. Dev Dyn. 2008;237(9):2554–65. 10.1002/dvdy.21680 . 18729213

[30] Novak AE, Taylor AD, Pineda RH, Lasda EL, Wright MA, Ribera AB. Embryonic and larval expression of zebrafish voltage-gated sodium channel alpha-subunit genes. Dev Dyn. 2006;235(7):1962–73. . 16615064 10.1002/dvdy.20811

[31] Kawahara A, Nishi T, Hisano Y, Fukui H, Yamaguchi A, Mochizuki N. The sphingolipid transporter spns2 functions in migration of zebrafish myocardial precursors. Science. 2009;323(5913):524–7. 10.1126/science.1167449 . 19074308

[32] Behra M, Cousin X, Bertrand C, Vonesch JL, Biellmann D, Chatonnet A, et al Acetylcholinesterase is required for neuronal and muscular development in the zebrafish embryo. Nature neuroscience. 2002;5(2):111–8. . 11753420 10.1038/nn788

[33] Kishore S, Fetcho JR. Homeostatic regulation of dendritic dynamics in a motor map in vivo. Nature communications. 2013;4:2086 10.1038/ncomms3086 23803587 PMC3702161

[34] Paradis S, Sweeney ST, Davis GW. Homeostatic control of presynaptic release is triggered by postsynaptic membrane depolarization. Neuron. 2001;30(3):737–49. . 11430807 10.1016/s0896-6273(01)00326-9

