# Supplementary figures and images for: Improved Long-Term Imaging of Embryos with Genetically Encoded α-Bungarotoxin
Source: PLoS One. 2015 Aug 5;10(8):e0134005. doi: 10.1371/journal.pone.0134005 (PMC4526548; doi:10.1371/journal.pone.0134005)

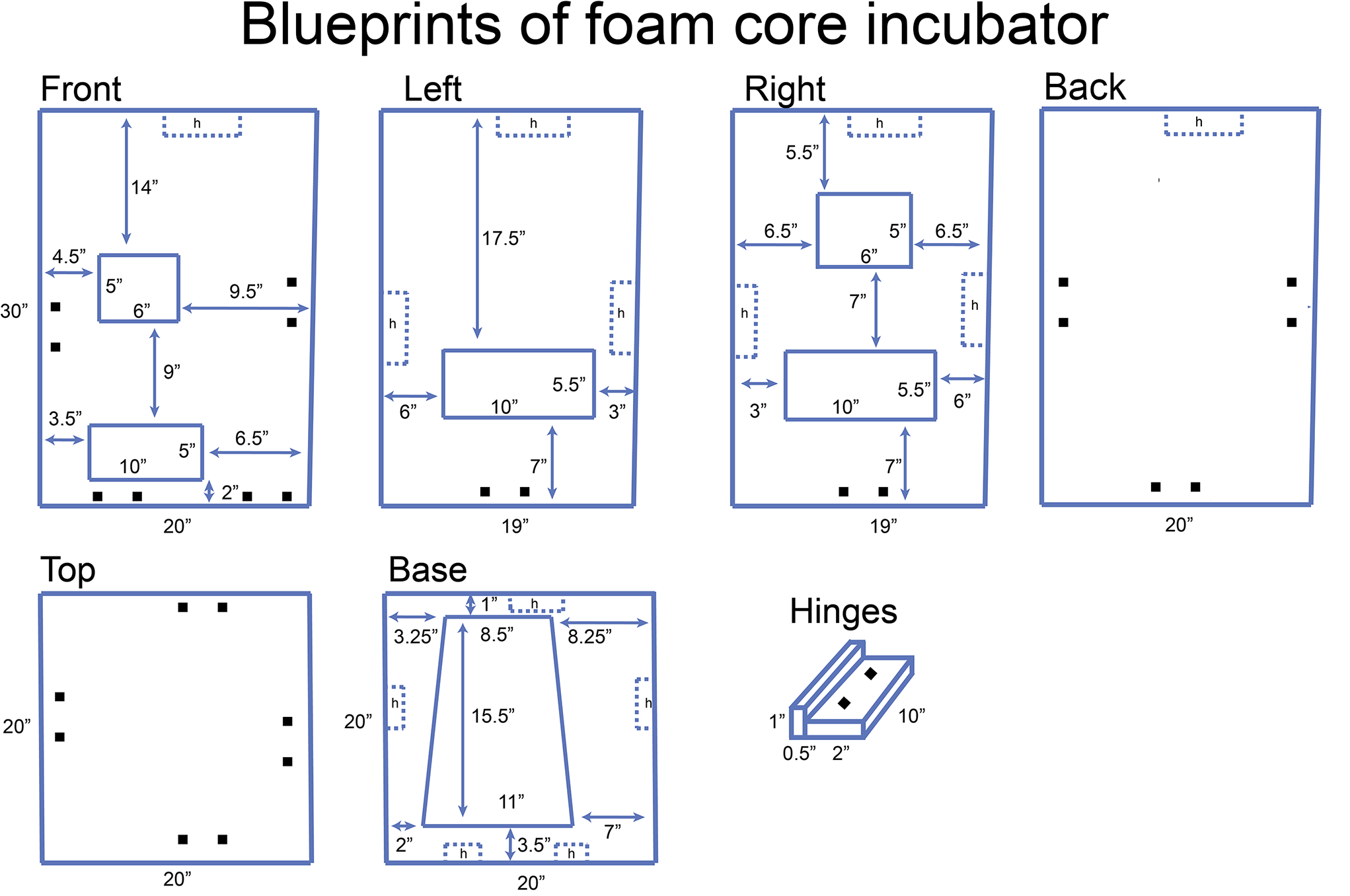

Supplement: S1 Fig — Panels were assembled by gluing one side of each hinge to a panel (locations depicted by dash rectangles containing ‘h’) and secured to the corresponding neighbor panel with nuts and screws inserted into holes drilled in foam core panels and hinges (depicted as black dots). (TIF) [file pone.0134005.s001.tif]

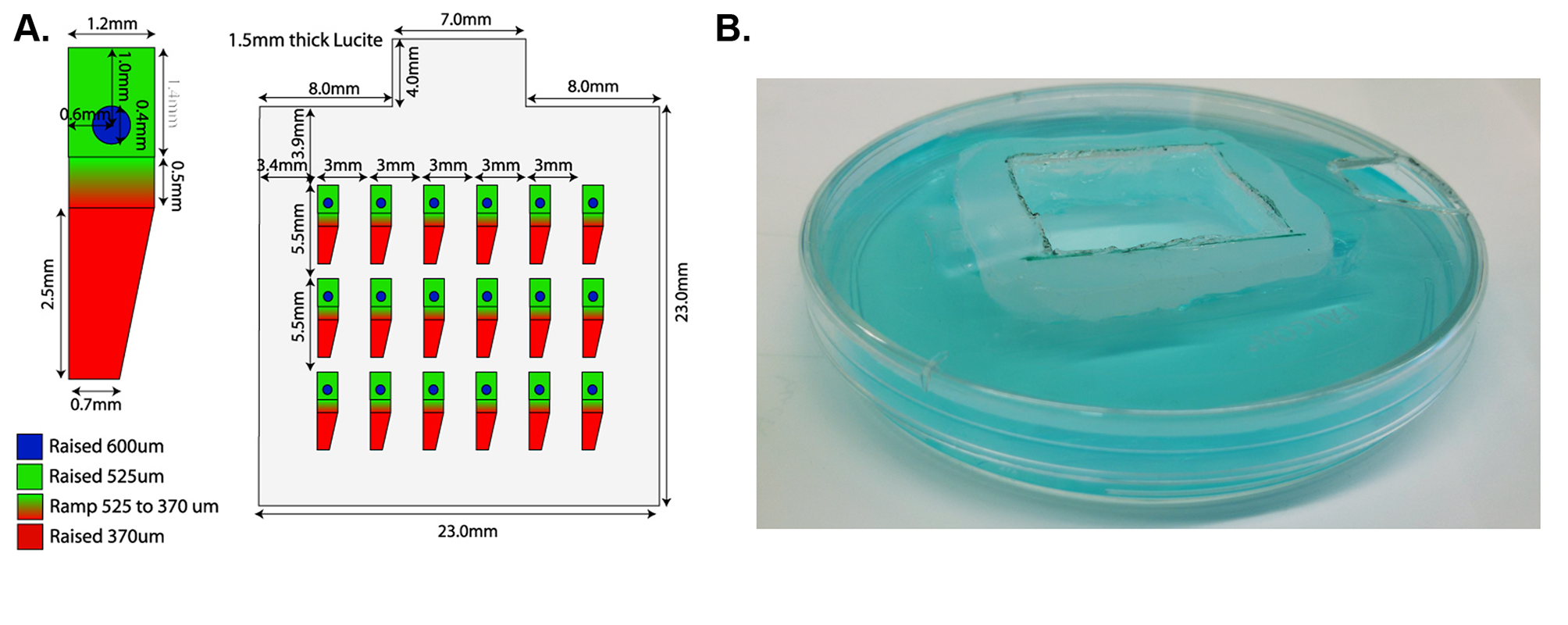

Supplement: S2 Fig — (A) Mold design customized so that embryo’s yolk sits in dimpled depression and tail can extend along a graded ramp of agarose. The graded ramp maintains more of the fish in the same focal plane. (B) Image of “skylight” petri dish used for long-term imaging. (TIF) [file pone.0134005.s002.tif]

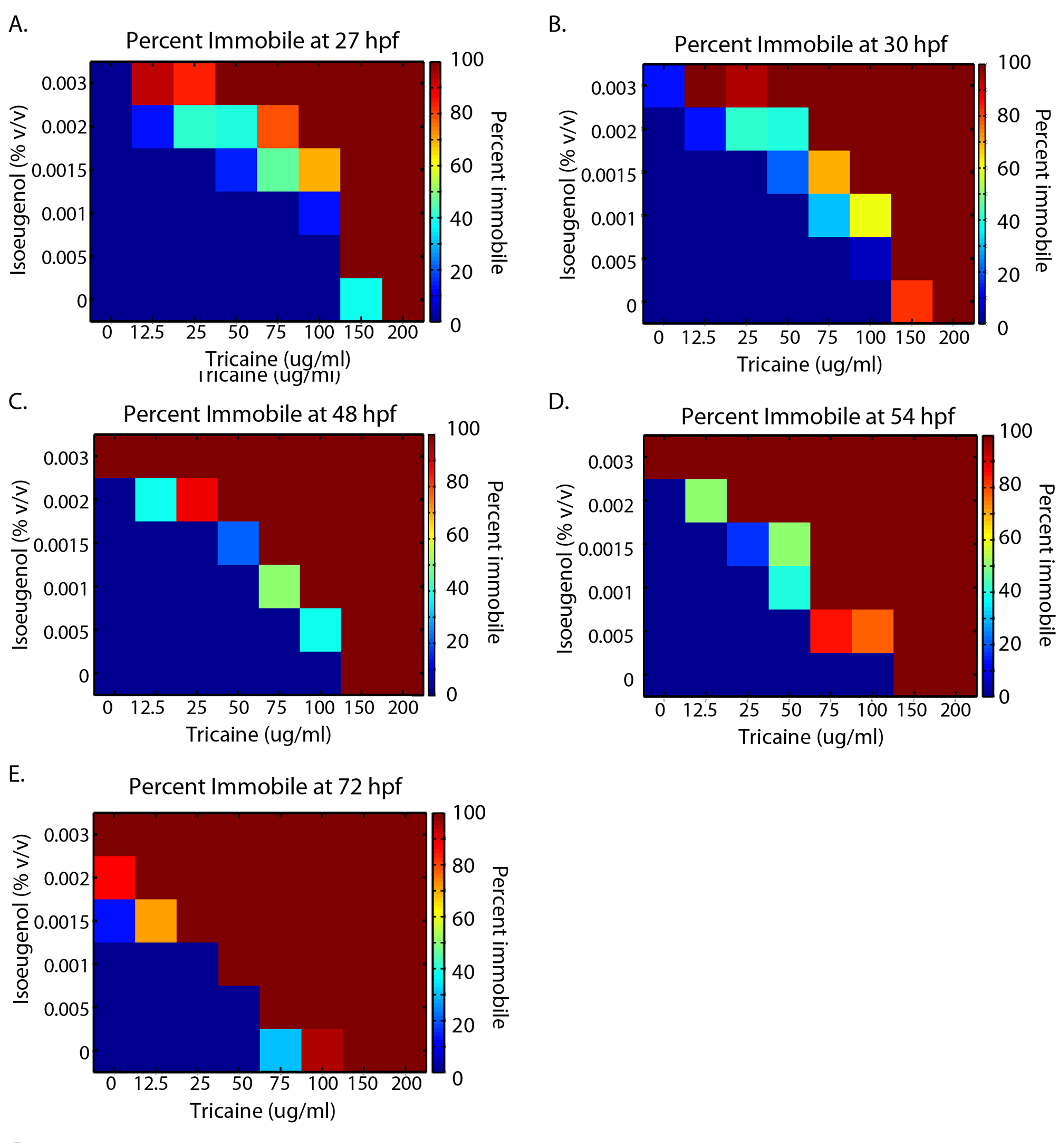

Supplement: S3 Fig — (A-E) Heat maps of percent immobile for 48 combinations of tricaine (0–200 μg/ml) and isoeugenol (0–0.003% v/v). Embryos were dechorionated and soaked starting at 24 hpf. Embryos were assayed for immobility at 27, 30, 48, 54, and 72 hpf. (TIF) [file pone.0134005.s003.tif]

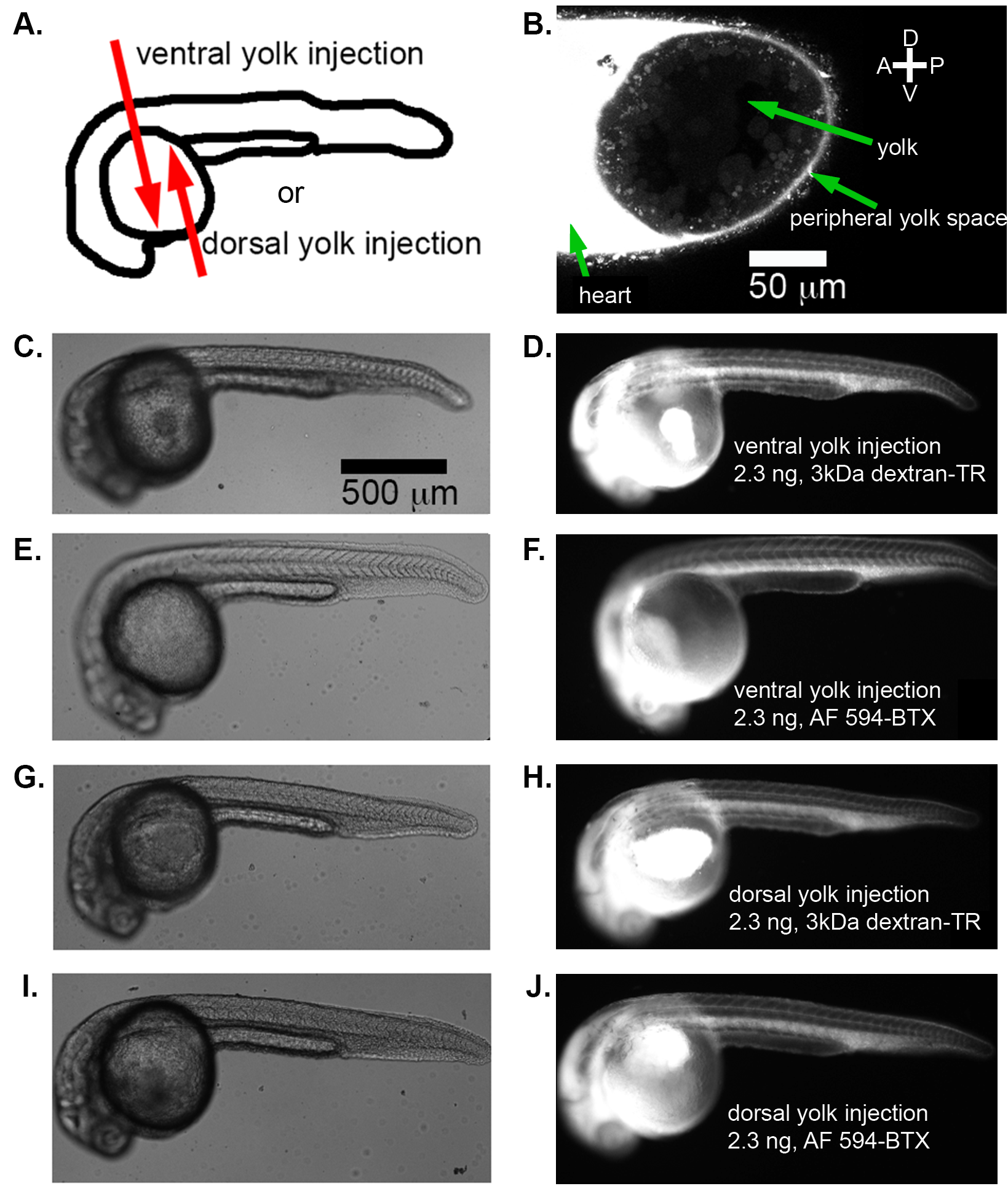

Supplement: S4 Fig — (A) Two injection strategies explored for α-bungarotoxin protein injection: either into the ventral side of the yolk or the dorsal side of the yolk of zebrafish embryos at 24 hpf. (B) Fluorescence from 2.3 ng Alexa-Fluor 594 conjugated α-bungarotoxin injected into the ventral yolk imaged by laser-scanning confocal microscopy. The peripheral yolk space appears continuous with the fluid entering the heart. DIC images (left) and fluorescent images (right) of representative embryos receiving ventral yolk injections of 2.3 ng 3 kDa dextran-Texas red (C-D), ventral yolk injections of 2.3 ng Alexa-Fluor 594 conjugated α-bungarotoxin (E-F), dorsal yolk injections of 2.3 ng 3 kDa dextran-Texas red (G-H), and dorsal yolk injections of 2.3 ng Alexa-Fluor 594 conjugated α-bungarotoxin (I-J). Scale bar in (C) applies for (C-J). (TIF) [file pone.0134005.s004.tif]
