# Supplementary material for: Improved Long-Term Imaging of Embryos with Genetically Encoded α-Bungarotoxin
Source: PLoS One. 2015 Aug 5;10(8):e0134005. doi: 10.1371/journal.pone.0134005 (PMC4526548; doi:10.1371/journal.pone.0134005)
Supplement: S1 Text — Text file containing the sequence of the synthetic α-bungarotoxin gene. Extended protocol for long-term imaging. (DOCX) [file pone.0134005.s006.docx]

**Supplemental Text S1**

Swinburne IA, Mosaliganti KR, Green AA, and Megason SG (2015)

**Sequence of synthetic α-bungarotoxin gene**

GenBank accession number KT279887

ATGAAGACCCTGCTGCTGACCCTGGTGGTGGTGACAATCGTGTGCCTGGACCTGGGATACACCATCGTGTGCCACACCACCGCCACCAGCCCTATCAGCGCTGTGACCTGCCCTCCTGGCGAGAACCTGTGCTACAGAAAGATGTGGTGCGACGCCTTCTGTAGCAGCAGAGGCAAGGTGGTCGAGCTGGGATGCGCCGCTACCTGCCCTAGCAAGAAACCTTACGAAGAGGTGACCTGCTGTAGCACCGACAAGTGCAACCCTCACCCTAAGCAGCGCCCTGGATGA

Sequence of α-bungarotoxin gene in expression plasmid

tcagccatcctaggcctatttaggtgacactatagaagagtactaatacgactcactatagggagaagctacttgttctttttgcaGGATCCGCCACCATGAAGACCCTGCTGCTGACCCTGGTGGTGGTGACAATCGTGTGCCTGGACCTGGGATACACCATCGTGTGCCACACCACCGCCACCAGCCCTATCAGCGCTGTGACCTGCCCTCCTGGCGAGAACCTGTGCTACAGAAAGATGTGGTGCGACGCCTTCTGTAGCAGCAGAGGCAAGGTGGTCGAGCTGGGATGCGCCGCTACCTGCCCTAGCAAGAAACCTTACGAAGAGGTGACCTGCTGTAGCACCGACAAGTGCAACCCTCACCCTAAGCAGCGCCCTGGATGATCTGCAGTAAGAATTCATACGTATCCGGAACCGGTgatccagacatgataagatacattgatgagtttggacaaaccacaactagaatgcagtgaaaaaaatgctttatttgtgaaatttgtgatgctattgctttatttgtaaccattataagctgcaataaacaagttaacaacaacaattgcattcattttatgtttcaggttcagggggaggtgtgggaggttttttgatatcccgggtttaaacgcatgcagat

sp6 promoter atttaggtgacactatagaagag

T7 promoter taatacgactcactataggga

α-bungarotoxin ORF

ATGAAGACCCTGCTGCTGACCCTGGTGGTGGTGACAATCGTGTGCCTGGACCTGGGATACACCATCGTGTGCCACACCACCGCCACCAGCCCTATCAGCGCTGTGACCTGCCCTCCTGGCGAGAACCTGTGCTACAGAAAGATGTGGTGCGACGCCTTCTGTAGCAGCAGAGGCAAGGTGGTCGAGCTGGGATGCGCCGCTACCTGCCCTAGCAAGAAACCTTACGAAGAGGTGACCTGCTGTAGCACCGACAAGTGCAACCCTCACCCTAAGCAGCGCCCTGGATGA

SV40 poly(A) sequence

gatccagacatgataagatacattgatgagtttggacaaaccacaactagaatgcagtgaaaaaaatgctttatttgtgaaatttgtgatgctattgctttatttgtaaccattataagctgcaataaacaagttaacaacaacaattgcattcattttatgtttcaggttcagggggaggtgtgggaggtttttt

**Long-term imaging**

A blue print for building a foam core chamber is presented in Fig. S1 and was assembled using 30x20x0.5 inch Elmers foam core board (Utrecht Art Supplies), M6 nylon flat washers (Amazon), M6-1 nylon hex nuts (Amazon), M6-1 nylon thumb screws (Amazon), and Gorilla glue (Amazon). The incubator was maintained at 28.5°C with a 225 Watt Cabinet IncuKit, Incubator Thermostat Fan Heater (Incubator Warehouse). The embryo was placed on top of an agarose mount set in a petri dish containing Danieau buffer, 0.001% methylene blue, and 1% penicillin and streptomycin. The agarose mount was generated by pouring 1.5% molten agarose in Danieau buffer and then setting a custom made Lucite mold atop the agarose (schematic of Lucite lateral mount mold (Fig. S2A)). A skylight window was made in the lid of the petri dish (Fig. S2B) by carving a window in the plastic and then sealing a depressed 43 x 50 mm coverslip (Electron Microscopy Sciences) with silicone glue (ALL-GLASS AQUARIUM). Images were acquired with an upright Olympus MVX10 Macroview (Olympus) using either a 2x MVX plan apochromat objective. Distilled water was added every 24 hours to compensate for evaporation. Images were acquired every 2 seconds using µ-Manager 1.4 [[1](#_ENREF_1)]. Acquired images were processed using Fiji [[2](#_ENREF_2)] and the movement index was calculated as the difference between each image and its subsequent image in the time-series normalized to the average difference in the first 2,000 time points.

**Supplemental References**

1. Edelstein A, Amodaj N, Hoover K, Vale R, Stuurman N. Computer Control of Microscopes Using µManager: John Wiley & Sons, Inc.; 2010.

2. Schindelin J, Arganda-Carreras I, Frise E, Kaynig V, Longair M, Pietzsch T, et al. Fiji: an open-source platform for biological-image analysis. Nature methods. 2012;9(7):676-82. doi: 10.1038/nmeth.2019. PubMed PMID: 22743772; PubMed Central PMCID: PMC3855844.
